# Emotional Egocentricity Bias Across the Life-Span

**DOI:** 10.3389/fnagi.2016.00074

**Published:** 2016-04-26

**Authors:** Federica Riva, Chantal Triscoli, Claus Lamm, Andrea Carnaghi, Giorgia Silani

**Affiliations:** ^1^International School for Advanced Studies (SISSA-ISAS)Trieste, Italy; ^2^Department of Basic Psychological Research and Research Methods, Faculty of Psychology, Social, Cognitive and Affective Neuroscience Unit, University of ViennaVienna, Austria; ^3^Department of Psychology, University of GothenburgGothenburg, Sweden; ^4^Department of Life Sciences, University of TriesteTrieste, Italy; ^5^Department of Applied Psychology: Health, Development, Enhancement and Intervention, Faculty of Psychology, University of ViennaVienna, Austria

**Keywords:** emotional egocentricity bias, empathy, aging, adolescence, life-span

## Abstract

In our daily lives, we often have to quickly estimate the emotions of our conspecifics in order to have successful social interactions. While this estimation process seems quite easy when we are ourselves in a neutral or equivalent emotional state, it has recently been shown that in case of incongruent emotional states between ourselves and the others, our judgments can be biased. This phenomenon, introduced to the literature with the term Emotional Egocentricity Bias (EEB), has been found to occur in young adults and, to a greater extent, in children. However, how the EEB changes across the life-span from adolescence to old age has been largely unexplored. In this study, we recruited 114 female participants subdivided in four cohorts (adolescents, young adults, middle-aged adults, older adults) to examine EEB age-related changes. Participants were administered with a recently developed paradigm which, by making use of visuo-tactile stimulation that elicits conflicting feelings in paired participants, allows the valid and reliable exploration of the EEB. Results highlighted a U-shape relation between age and EEB, revealing enhanced emotional egocentricity in adolescents and older adults compared to young and middle-aged adults. These results are in line with the neuroscientific literature which has recently shown that overcoming the EEB is associated with a greater activation of a portion of the parietal lobe, namely the right Supramarginal Gyrus (rSMG). This is an area that reaches full maturation by the end of adolescence and goes through an early decay. Thus, the age-related changes of the EEB could be possibly due to the life-span development of the rSMG. This study is the first one to show the quadratic relation between age and the EEB and set a milestone for further research exploring the neural correlates of the life-span development of the EEB. Future studies are needed in order to generalize these results to the male population and to explore gender differences related to the aging of socio- emotional processes.

## Introduction

General and social psychology have long acknowledged that human’s decisions and behaviors are influenced by egocentric tendencies (Greenwald, 1980; Nickerson, 1999; Royzman et al., 2003). Observable early in life, they can affect different socio-cognitive processes, such as Theory of Mind (ToM) and visual perspective taking (PT).

In his seminal work, Piaget (Piaget and Inhelder, 1956), relied on the famous task of the “three mountains” and observed that until 7 years old, children were unable to take another person’s visual perspective, producing ego-biased responses. In this task, children were asked to look at a display with three mountains and to indicate what view was seen by an observer (a doll) located in a different position. Since then, several studies have shown that children have difficulties in detaching from their own perspectives and beliefs to attribute different mental states to or to take the perspectives of another person (e.g., Wimmer and Perner, 1983; Baron-cohen et al., 1985; Wellman and Woolley, 1990; Robinson and Mitchell, 1995; Southgate et al., 2007; Surtees and Apperly, 2012).

Egocentric biases in PT and ToM have also been fatherly investigated at different ages across the life-span. Recent studies showed that ToM continues to improve until late adolescence where egocentric tendencies are still detectable. For example, Dumontheil et al. (2010) measured visual PT in four groups, ranging from older children to younger adults, and found an improvement in the performances until early adulthood. In another recent article by Bosco et al. (2014), 80 pre-adolescents and adolescents were screened with a broad set of ToM tasks and structured interviews, revealing an earlier maturation of the ability to reason about own mental states than reasoning about those of the others, likely resulting from the adoption of egocentric viewpoint when reasoning about others.

Cognitive processes are affected by egocentric biases in adulthood as well. For example, Birch and Bloom (2007) showed that adults’ ability to reason about another person’s belief on an event is compromised by their own knowledge about that specific event, if the task is sufficiently challenging. Furthermore, in a series of studies, Keysar and colleagues (Keysar et al., 2000; Epley et al., 2004) observed that when participants have to follow the instructions of a person with a different visual perspective regarding how to displace objects in a shelf, they committed more self-perspective related errors. Similarly, Surtees and Apperly (2012) investigated visual-PT in a computer-based task where participants’ aim was to provide judgments during time-pressure condition about the number of dots on a wall seen by an avatar. Importantly, the avatar could have either the same perspective of the participants or a different visual perspective. Results showed that participants were slower and less accurate in judging avatar’s perspective when this was different by their own perspective.

Finally, the ability to control the cognitive egocentrism has been observed to decline in the elderly. Inagaki et al. (2002) observed for example that older adults performed worse than younger adults in a visual PT task, with the former group producing more egocentric responses than the latter. In another relevant study, Bailey and Henry (2008) recorded worse performances in old compared to young adults in a ToM false-belief task. Importantly, the performance in this task was found to be associated with a decline in inhibiting self-perspective which led to more egocentric responses in older than in young adults.

Besides these cognitive processes, egocentric tendencies can also occur in the emotional domain, for example when we have to deal with the emotions of others, being ourselves in an opposite emotional state (e.g., when we are happy because our article just got accepted, while a colleague sitting next to us is disappointed because his article just got rejected).

Similarly to cognitive egocentrism, the investigation of the development of emotional egocentricity has revealed greater bias in younger compared to older children and adults. For example, Repacholi (Repacholi and Gopnik, 1997), employing a suitable task for infants, showed different levels of egocentric tendencies in infants between 14 and 18 months-old, being the latter better than the former to inhibit own food preferences and thus to give the experimenter the food toward which he before expressed positive emotions.

In adults, this phenomenon has been investigated only by a recent study in which a new paradigm enabling the valid and reliable exploration of the Emotional Egocentricity Bias has been developed (EEB; Silani et al., 2013). In this paradigm, pleasant or unpleasant feelings were induced in the participants (in pairs) by using conjoint visuo-tactile stimulation. Participants were then asked to empathize with and to judge other participant’s emotions. Importantly, the emotions of the two participants could be either aligned or in opposite. Researchers observed that empathic judgments of the other participant’s emotional state were significantly affected by their own current emotional state, giving rise to an egocentrically biased evaluation. Notably, the EEB was associated with a greater activation of a portion of the parietal lobe, namely the right Supramarginal Gyrus (rSMG), an area that reaches full maturation by the end of adolescence and go through an early decay (Sowell et al., 2003).

Employing a paradigm closely matched to the one used by Silani et al. (2013), two following studies have shown that EEB is stronger in children aged from 7 to 12 years old compared to adults (Steinbeis et al., 2014; Hoffmann et al., 2015). Importantly, Steinbeis et al. (2014) observed group differences in the same portion of rSMG associated to the overcoming of the EEB, suggesting a relationship between the age-related changes of the EEB and the functional and structural maturation of this brain area.

Taken together, previous studies provided robust evidence for the existence of egocentric biases in the cognitive and the visuo-perceptive domains across the life-span, with stronger egocentric tendencies especially in children, but also in adolescents and older adults compared to young adults. By contrast, little is known about the development of egocentric bias in the emotional domain. Indeed, while, as previously mentioned, EEB has been shown to occur at a greater extent in children than in adults, to the best of our knowledge, no study has so far explored how emotional egocentric tendencies develop from adolescence to the old age. The present study, thus, aimed at filling this gap, by investigating the age-related changes of the EEB from adolescence to old age. To address this question, the newly established paradigm from our group (Silani et al., 2013) able to induce a quantifiable EEB was performed by four cohorts in a cross-sectional study. The four cohorts specifically comprised adolescents, young adults, middle-aged adults and older adults. According to previous literature about life-span development of cognitive egocentrism, we hypothesized adolescents and older adults to be characterized by a higher EEB with respect to younger and middle-aged adults, resulting in a U-shaped relations between EEB and age.

## Materials and Methods

### Participants

One hundred fourteen female individuals (age = 13–78) took part in the study, performed at the Scuola Internazionale Superiore di Studi Avanzati (SISSA, Trieste, Italy). One participant was discarded since her age was missing. Therefore, analyses were performed on 113 participants in total. All participants were right-handed (Edinburgh Handedness Inventory; Oldfield, 1971), had normal or corrected-to-normal vision, and reported no past or present neurological or psychiatric disorder. Only female participants were recruited and for two main reasons: (a) consistency with our previous work in which only females were tested (Silani et al., 2013); and (b) because of documented gender differences in empathy and socio-affective skills (Schulte-Rüther et al., 2008; Tomova et al., 2014). Four groups were established according to age^1^ (see Table 1) for the categorical analyses. Years of education were also taken into account.

**Table 1:** **Demographic data for the 113 female participants**.

	*N*	Age range	Mean age (SD)	Mean years of education (SD)
Adolescents	28	13–17	15.7 (1.5)	10.8 (1.5)
Young adults	30	20–30	24.9 (2.3)	17.8 (1.8)
Middle-aged adults	29	33–59	43.9 (6.9)	14.93 (3.9)
Older adults	26	63–78	70.6 (4.9)	10.9 (4.4)

Older adults underwent a brief neuropsychological screening to control for initial stages of neurodegenerative disease. The Italian version of the Mini Mental State Examination (Magni et al., 1996) was thus administered. None of the older participants fell under the cut-off (≥24) for cognitive decline.

This study was conducted in accordance with the Declaration of Helsinki (1983) and local guidelines of the SISSA. All participants gave written informed consent prior to the experiment. Written informed *parental* consent was required for adolescents. Subjects were paid 15 euros for their participation.

### Stimuli and Procedure

The newly-established task (see Silani et al., 2013; Lamm et al., 2015 for further details) was employed to measure the EEB in the four age groups.

Each experimental session comprised a pair of participants belonging to the same cohort and unknown to each other. The two participants were to sit at two different desks in the same room, back to back, in front of a touch screen (800 × 600 pixels resolution, 15 inch screen, viewing distance ~40 cm) and with their left hand under a curtain to prevent them to see it. By means of either pleasant or unpleasant visuo-tactile stimulations, transient emotional states were induced in the subjects. Visuo-tactile stimulations consisted of presenting on the screen a picture showing either an agreeable or a disagreeable object (e.g., rose or worm) in association with the stroke of the participants’ hidden hand with a material resembling the object depicted, able to induce pleasant or unpleasant emotions. Together with the picture of the object touching the participant, a picture of the object stroking the paired participant was presented on the screen. The labels “You” and “Other” were shown above the pictures indicating the correspondence between the objects and the target of the stimulation. Stimulations could be either congruent or incongruent in respect to the emotional state elicited in the other person. After the tactile stimulation (3 s duration), participants were instructed to assess the pleasantness of the stimulations on a rating scale ranging from −10 to +10 by pressing with their right index the touch screen, within maximally 3 s. In the *self-judgment* condition, participants had to evaluate their own feelings, while in the *other-judgment* condition they had to judge other participant’s feelings. Each run consisted of 40 pseudorandomized trials, with 20 pleasant (10 congruent/10 incongruent) and 20 unpleasant (10 congruent/10 incongruent) visuo-tactile stimuli. Before performing the actual task, subjects underwent a training phase which was ending only after the participant had fully understood the instructions of the task. Once this condition was satisfied, each subject’s rating was retained.

### Computation of the EEB

As in Silani et al. (2013), EEB was calculated by computing the difference between other-related ratings during incongruent vs. congruent trials. We predicted these empathic judgments to be biased by one’s own emotional state, especially in adolescents and older adults. From this difference, to control for unspecific aspects such as incongruence detection or stimulus conflict, we subtracted the same difference during self-related trials. The EEB value (with higher values indicating higher interference by one’s own emotional state on rating the other’s) was computed for each subject and employed for all the subsequent analysis.

As for our original work (Silani et al., 2013), EEB values didn’t undergo a statistical procedure for excluding outliers, since the same nature of the task implies in certain conditions an altered estimation and thus deviant values are expected, especially in the more external group.

To test whether the EEB differs according to the age of the participants, we first performed a one-way ANOVA on the EEB values employing the cohort (adolescents, young adults, middle-aged adults, older adults) as the between-group factor. Given our hypothesis regarding the U-shaped development of the EEB, we tested for the significance of both a linear and a quadratic trend. As a second step, a regression analysis between the total EEB score and the age of the participants was performed. In a third step, all the above-mentioned analyses were run controlling for the years of education. Analyses were performed using the IBM SPSS statistics Software, version 20.0.

For each linear and quadratic model the relative AIC (Akaike’s information criteria) index was also computed using: http://graphpad.com/quickcalcs/AIC1.cfm.

## Results

**Figure 1:**
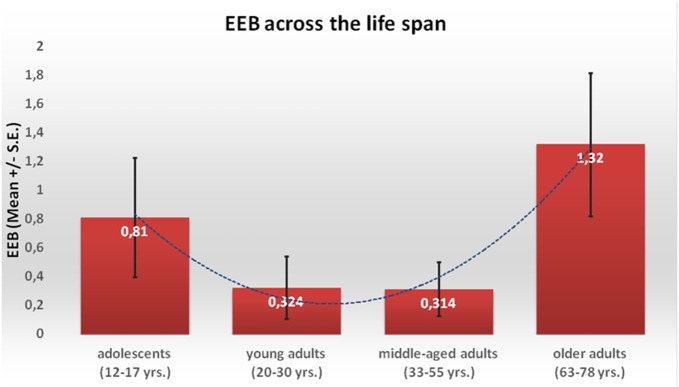
**Representation of the Emotional Egocentricity Bias (EEB) in the four cohorts (mean and SE)**.

Results from the one-way ANOVA showed that the linear trend was not significant (*F*_(1,109)_ = 1019; *p* = 0.315; *η*^2^ = 0.008), whereas the quadratic trend was statistically significant (*F*_(1,109)_ = 4.745; *p =* 0.03; *η*^2^ = 0.04). The EEB score (Figure 1) decreased from the adolescents (*M* = 0.81, *SE* = 0.42) to the young adults (*M* = 0.32, *SE* = 0.22) and the middle-aged adults group (*M* = 0.31, *SE* = 0.19), and increased thereafter in the older adults (*M* = 1.35, *SE* = 0.52).

**Figure 2:**
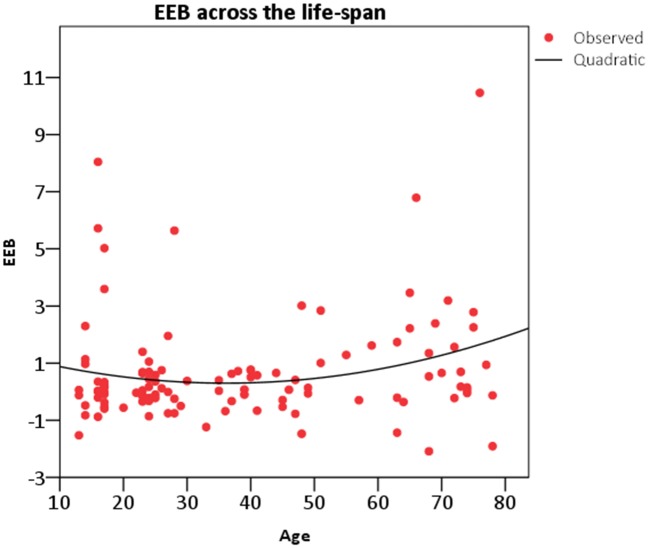
**Representation of the quadratic relation between age and EEB observed with the non-linear regression model**.

To test the same hypothesis in a different manner, the EEB was regressed on the age of participants in a non-linear regression model. Again, the linear term was not significant, (*R*sq = 0.02, *F*_(1,111)_ = 2.62, *p* = 0.11, *R* = 0.152, AIC = 142.47), whereas the quadratic term (*R*sq = 0.05, *F*_(2,110)_ = 2.64, *p* = 0.076, *R* = 0.214, AIC = 141.91) fell short off of significance. The EEB (Figure 2) decreased from the adolescents to the young adults, it remained stable in the middle-aged adults and increased thereafter in the older adults. Comparison of the two AIC (difference in AICc = 0.56, information ratio = 1.32) also confirmed that the quadratic model is better than the linear model to explain the relationship between age and EEB.

In sum, the magnitude of the EEB displayed a quadratic association with participants’ age, both when age was treated as a categorical variable and as a continuum variable.

However, an alternative hypothesis would argue that participants’ level of education could account for this pattern of results. Indeed, participants’ education was significant associated with participants’ age, both in a linear trend *R*^2^ = 0.05, *F*_(1,111)_ = 5.54, *p* = 0.020, and in a quadratic trend *R*^2^ = 0.32, *F*_(2,110)_ = 25.870, and the quadratic trend explained 27% of the variance more than the linear trend. This pattern of result revealed that the education increased as the age increased, peaked in the middle-age, and decreased thereafter. In order to rule out the alternative hypothesis, we first checked whether the EEB was associated with the level of education. The EEB score was regressed on participants’ level of education in a non-linear model. Neither the linear *R*^2^ = 0.005, *F*_(1,111)_ = 0.58, *p* = 0.45, nor the quadratic term *R*^2^ = 0.03, *F*_(2,110)_ = 1.51, *p* = 0.23 were significant. Second, the EEB score was analyzed by an ANCOVA with cohort as between-participants factor and participants’ level of education as covariate. The covariate was not significantly associated with the EEB score, *F*_(1,113)_ = 0.75, *p* = 0.39, partial *η^2^* = 0.007. Importantly, the quadratic trend was significant (*p* = 0.031), even after controlling for participants’ level of education. Third, participants’ age was regressed on their level of education (quadratic trend) and the residual were saved. In a non-linear regression model, residuals (age) were entered as the predictor and the EEB as the dependent variable. The linear association between the age and the EEB was marginally significant (*R*^2^ = 0.03, *F*_(1,111)_ = 3.65, *p* = 0.06, AIC = 141.46) whereas the quadratic association between age and EEB was significant (*R*^2^ = 0.07, *F*_(2,110)_ = 4.08, *p* = 0.02, AIC = 139.14) and explained 4% of the variance more than the linear model. Even in this case the comparison of the AIC (difference in AICc = 2.32, information ratio = 3.19) relative to the two models bore out our hypothesis. Taken together these results rule out the alternative hypothesis that the quadratic association between age and EEB was mainly driven by participants’ level of education.

## Discussion

In the present study, we investigated how the EEB evolves across the life-span, from adolescence to older adulthood, in a sample of 113 female participants. By eliciting transient contrasting emotional states in paired participants, we measured to what extent individuals are affected by their own emotional states when evaluating those of the others. The task was administered to four groups of different age and specifically to adolescents (age range: 13–17), younger adults (age range: 20–30), middle-aged adults (age range: 33–59) and older adults (age range: 63–78).

In order to examine age-related differences in emotional egocentric tendencies, an index of the EEB was computed for each participant and then employed for implementing group comparison analysis. Results confirmed our initial hypothesis showing a general increase of the EEB in the extreme age groups (adolescents and older adults) as demonstrated by the significant quadratic relationship between age and EEB. This U-shaped relation between age and EEB was significant both when age was treated as a categorical variable and as a continuum variable and irrespective of the level of education of the participants.

Overcoming EEB is a complex process that relies on different abilities, such as on the ability to recognize emotional states, to distinguish between our own and other-related representations, and to inhibit self-perspective/emotional states in order to focus on the socially relevant information (other). Previous literature on the development of these abilities are in line with the results of the present study.

In a study by Keulers et al. (2010), adolescent participants (13–20 years old) were required to take another person’s perspective in an emotionally arousing situation and to evaluate her/his emotional state. A linear decreasing across age for affective mentalizing speed emerged, suggesting a linear development across adolescence of affective ToM. In another study by Sebastian et al. (2012), adolescents and adults were administered a different vignette-based affective ToM task where they had to choose the appropriate ending vignette representing a character’s reaction to her/his companion’s emotional state. While no differences emerged in reaction times, adolescents were found to make more mistakes than adults. Employing another affective ToM task, Vetter et al. (2013) asked participants (age range: 12–22) to choose the adjective that describes best the affective mental state depicted by an actor in a video-clip. The authors found a positive strong correlation between performance and age indicating an affective ToM development until late adolescence. In addition, in the same study the authors analyzed the contribution of different Executive Functions measures to affective ToM ability and found that the age-related improvement in the affective ToM was significantly more predicted by the improvement of inhibition ability than by shifting and updating ability. Given that overcoming the EEB implies self-perspective to be inhibited, this result is particularly in line with our findings.

Results on the decline of socio-cognitive abilities associated to the EEB have been also observed at the other extreme of the life-span. For example, it has been shown that recognizing emotional facial expressions for older adults is not as easy as for younger ones (Phillips et al., 2002). In a different study, Bailey and Henry (2008) administered a false-belief task to young (age range: 18–26) and older adults (age range: 62–82), manipulating the amount of self-perspective inhibition needed to solve different problems. Results showed that older adults performed worse than young adults just in the high-inhibition trials, suggesting an increasing difficulty in detaching from self-perspective with aging. Moreover, Ze et al. (2014) investigated trait affective empathy in young and older adults by administering the Multifaceted Empathy Task (MET; Dziobek et al., 2008) and found older adults to score higher in empathic concern (“how strongly they feel for the person of the story) and personal involvement (“how strongly they feel affected by the presented story”). Although this might seem in contrast with studies presented above, high concern and personal distress may result from reduction in self-other distinction as pointed out by the authors. In addition, a positive correlation between these two measures and performance on a response inhibition task was found. Again, a recent meta-analysis (Henry et al., 2013) conducted on different tasks testing ToM ability in older adults confirmed that elderly people encounter more difficulties than younger adults in understanding complex emotional and mental states experienced by others.

Taken together, these studies suggest that complex socio-cognitive abilities continue to develop until late adolescence and decline early with aging, proving compelling evidence for the age-related trajectory of the EEB, revealed here for the first time.

A possible explanation of the EEB trajectory may be the underlying development of the executive functions. Indeed, Hoffmann et al. (2015) tested EEB development in children ranging from 7 to 13 and found EEB development to be mediated by the ability of conflict processing. Notably, a quadratic relations between age and conflict processing has been already reported in several studies, with children, adolescents and older adults showing worse performances (Li et al., 2009; Hämmerer et al., 2010). Moreover, Huizinga et al. (2006) observed that the ability of attentional shifting continues to develop through adolescence, while Schnitzspahn et al. (2013) found shifting to decrease with age. Therefore, there is the possibility that the age-related changes of the EEB may be partially due to the development of some components of the executive functions, as conflict monitoring and shifting. Future studies are needed to further explore this relationship over the life-span.

Results observed in this study are also in line with the recent neuroscientific literature which suggests that a late maturation and early decay of the brain regions could be involved in the regulation of the egocentricity bias. In the seminal article by Silani et al. (2013), neuroimaging results revealed the EEB to be associated with the activation of a specific brain area, a portion of the parietal lobule corresponding to the Brodmann area 40, specifically the right SMG. Using Transcranial Magnetic Stimulation (TMS), the researchers provided evidence for a causal role of the rSMG in overcoming egocentric evaluations of other’s emotional states. Steinbeis et al. (2014) by exploring the neural basis of the EEB in children observed a lower activation of the rSMG compared to adults, and a decrease of EEB over age. Notably, studies focused on the structural development of the brain have observed a late maturation of specific parts of the parietal lobule (Giedd et al., 1999) and in particular of the SMG (Gogtay et al., 2004), area that seems to fully mature during adolescence. In addition, research on brain aging indicates a linear reduction of the gray matter volume during adulthood with a consistent drop after the seventh decade (Courchesne et al., 2000) that involves, together with the frontal lobe, the parietal lobe (Sowell et al., 2003).

Taken together, these studies suggest that the quadratic relation between age and EEB could be due to the quadratic relation between age and development and maturation of the rSMG. The results of this study set a milestone for exploring further this issue.

In conclusion, we were able to provide the first evidence that the emotional egocentric bias in female participants develops across the life-span following a U-shaped trajectory, with adolescents and older adults exhibiting a higher level of emotional egocentrism compared to younger and middle-aged adults. Further research is needed to clarify how this error in judging other emotions is actually associated to the structural and functional development of dedicated brain regions and to extend these observation to a male aging sample.

## Author Contributions

GS designed the research; CT performed the research; FR and AC analyzed the data; GS and FR wrote the article; AC, CL and CT provided critical revisions.

## Conflict of Interest Statement

The authors declare that the research was conducted in the absence of any commercial or financial relationships that could be construed as a potential conflict of interest.

## Footnotes

^1^– *28 adolescents, age range: 13–17 years old*. This group covers the puberty period. According to the American Psychological Association, APA. (2002), puberty in females starts around 10–12 years old and is complete about the age of 17. – *30 young adults, age range: 20–30 years old*. In this group participants’ have reached their full biological maturation (e.g., Sandu et al., 2014). – *29 middle-aged adults, age range: 33–59 years old*. With this group we cover a life period which has been so far largely under-investigated, that lies in the middle between individuals who have just become adults and individuals who are approaching the last life stage. – *26 elderlies, age range: 63–78 years old*. The fourth group covers the last life stage, marked by the important life event of retirement.
